# Does Anti-Müllerian hormone vary during a menstrual cycle? A systematic review and meta-analysis

**DOI:** 10.1186/s13048-022-01006-z

**Published:** 2022-07-01

**Authors:** Rasa Khodavirdilou, Marjaneh Pournaghi, Yeganeh Rastgar Rezaei, Khadijeh Hajizadeh, Lida Khodavirdilou, Farzin Javid, Kobra Hamdi, Mahnaz Shahnazi, Mohammad Nouri, Amir Fattahi, Matthias W. Beckmann, Ralf Dittrich

**Affiliations:** 1 Womenʼs Reproductive Health Research Center, Tabriz University of Medical Sciences, Tabriz, Iran; 2 Department of Medical Biotechnology, Faculty of Advanced Medical Sciences, Tabriz University of Medical Sciences, Tabriz, Iran; 3 Department of Midwifery, Faculty of Nursing and Midwifery, Tabriz University of Medical Sciences, Tabriz, Iran; 4 Faculty of Pharmacy, Tabriz University of Medical Sciences, Tabriz, Iran; 5 Department of Reproductive Biology, Faculty of Advanced Medical Sciences, Tabriz University of Medical Sciences, Tabriz, Iran; 6 Department of Obstetrics and Gynecology, Erlangen University Hospital, Friedrich-Alexander University of Erlangen–Nürnberg, Erlangen, Germany

**Keywords:** Anti mullerian hormone, Menstrual cycle, Fullicular phase, Luteal phase, Ovulation, Systematic review, Meta-analysis

## Abstract

**Objective:**

Numerous studies have indicated that the level of the Anti-Müllerian hormone (AMH), one of the main markers for the ovarian reserve, does not fluctuate throughout a menstrual cycle, while some studies have rejected this finding. The purpose of this systematic and meta-analysis study is to consensus on all contradictory studies that have measured AMH levels throughout the menstrual cycle and to investigate the exact extent of AMH variation in a cycle.

**Methods:**

The protocol for this meta-analysis was registered at PROSPERO before data extraction. Relevant studies were identified by systematic search in PubMed, ScienceDirect, Embase, Cochrane Library, and Google Scholar with no limitation on publication date. Longitudinal studies which have evaluated AMH levels in the follicular and luteal phases of an unstimulated (natural) menstrual cycle in healthy women without endocrinology or ovarian disorders were included. We used the JBI Critical Appraisal Checklist for assessing the quality of studies found eligible for meta-analysis.

**Results:**

A total of 11 studies involving 733 women with regular menstrual cycles were included. The results showed that the AMH level in the follicular phase was significantly higher than in the luteal phase (95% Cl = 0.11 [0.01 to 0.21]; *p* < 0.05) and it varies about 11.5% from the luteal phase. The analysis of studies which had also examined the ovulatory phase (*n* = 380) showed that the serum levels of AMH in the ovulatory phase (about 2.02 ng/ml) did not significantly vary compared to follicular (95% Cl = 0.11 [-0.10 to 0.33]; *p* = 0.30) and luteal (95% Cl = 0.06 [-0.08 to 0.20]; *p* = 0.43) phases.

**Conclusions:**

According to the results of this study, AMH levels differ between follicular and luteal phases which might be due to ovarian response to the gonadotropins. It seems the phase of AMH measurement needs to be considered for interpretation of the serum AMH test.

## Background

Anti-Müllerian hormone (AMH) is produced by ovarian granulosa cells, and its blood levels are low at birth and reach their maximum at puberty and reproductive age of females. However, AMH levels gradually decrease until they reach undetectable values in post-menopause [1–5]. Studies have documented that AMH plays a role in the inhibition of primary follicle utilization, FSH-dependent growth inhibition, and selection of preantral and small antral follicles [6].

Given the positive association of AMH levels with the primary antral follicle counts (AFC), this hormone is considered one of the main markers for ovarian reserve as well as ovarian response in controlled ovarian hyperstimulation (COH) [5]. Therefore, the serum levels of AMH are used to follow the age-related decrease in follicle counts [7]. Different methods are commercially available to measure blood levels of AMH. In the past, two AMH assay kits are mainly used for evaluating AMH concentration, including Immunotech assay (Beckman Coulter, Texas, USA) and the Diagnostic System Laboratories assay (DSL; Diagnostic System Laboratories, Texas, USA); however, studies have shown wide differences between the results of these kits [8–11]. Recently, a second-generation enzyme-linked immunosorbent assay (ELISA) called GEN II has been developed to measure AMH levels [12, 13].

Considering the importance of AMH levels in predicting the fertility capacity of women, especially in assisted reproductive technology (ART) cycles, different studies have been conducted to evaluate AMH fluctuations during a menstrual cycle to address how blood levels of AMH change between the follicular and luteal phases and how the results can be interpreted. In this regard, several studies reported that the AMH level does not alter during the cycle, and some have shown that its level varies during the menstrual cycle.

There is no consensus on the issue of whether the AMH levels vary throughout a menstrual cycle or not. Unfortunately, there are no specific guidelines for the time of AMH measurement and most physicians refer patients for serum AMH testing without considering the phase of the menstrual cycle. To the best of our knowledge, there is no meta-analysis study regarding the variation of AMH in a menstrual cycle. Considering the clinical importance of AMH levels in the evaluation of ovarian reserve and also choosing the therapeutic method for patients with ovarian failure (e.g. Platelet-rich plasma or hormone therapies), in this systematic review and meta-analysis study, we tried to address whether AMH levels fluctuate throughout the menstrual cycle or not.

## Methods

Preferable Reporting Items for Systematic Reviews and Meta-Analyses (PRISMA) guidelines were followed for reporting this systematic review and meta-analysis and followed a structured protocol settled among the authors before starting the literature search [14]. A protocol for this review study was registered in the PROSPERO database (ID: CRD42021282887).

### Search strategy

The present systematic review involved all published research articles that have investigated AMH fluctuations through the menstrual cycle. A systematic search in Google Scholar, PubMed, Sciencedirect, Embase, and Cochrane Library databases was performed with no limitation on the date of publication using a combination of the following search terms: “AMH,” OR “Anti Mullerian hormone” OR “Mullerian inhibiting factor” OR “Mullerian inhibiting substance”, AND “Menstrual cycle” OR “Ovulatory cycle” OR “Follicular phase” OR “Luteal phase” OR “Ovulation”. Cross-references were checked to assess if any relevant studies had been missed. Consequently, three researchers (R.K., M.P., Y.R.) accurately read and individually assessed all selected articles based on the inclusion/exclusion criteria.

### Eligibility criteria

Studies were included if they met the following criteria: 1) studies that reported AMH levels in both follicular and luteal phases of a menstrual cycle, 2) studies on women at non-stimulated (natural) menstrual cycle, and 3) studies that were written in the English language. Furthermore, articles with the following statuses were excluded: 1) reviews, commentary, conference proceedings, and theses, 2) studies that evaluated the AMH level at one phase of the menstrual cycle or two phases of different cycles, 3) studies on women with a stimulated cycle, 4) study on women with endocrinological and ovarian disorders such as polycystic ovaries syndrome (PCOS) and premature ovarian failure (POF), and 5) articles that lacked sufficient information for analysis.

### Study selection and data collection

Preferred Reporting Items for Systematic Review and Meta-Analysis (PRISMA) were followed. We included the studies that met the inclusion criteria and provided extractable information about the AMH change during the menstrual cycle. In the articles that met the criteria but had no extractable information for the meta-analysis, the authors were communicated via email. If the authors did not respond or the required data were not available, the articles were excluded from the study. For each study, the first author, year of publication, number of cases, mean age of the patients, mean ± standard deviation (SD) of AMH levels in two follicular and luteal phases in ng/ml unit (picomolar unit was converted to ng/ml using the conversion formula of 1 ng/ml = 7.14 pmol/l), AMH assay method, study plan, and patient recruitment strategy were extracted.

### Quality assessment

For quality assessment, the Joanna Briggs Institute (JBI) Critical Appraisal Checklist for Prevalence Studies was used to assess the methodological quality of the selected studies by two reviewers independently [15]. A consensus meeting resolved disagreements between the reviewers. Nine items were used to assess the risk of bias in each study evaluating the quality of the study in the domains of the study population, data collection, and data analysis. The risk of bias categories was judged by counting the results of the sections: > 70% was considered high quality/low risk, 50–70% as medium quality/moderate risk, and < 50% was considered as a high risk/low quality [16].

### Publication bias

To evaluate asymmetry, funnel plots were analyzed. Begg's rank correlation and Egger's linear regression tests were used to detecting possible publication bias. A two-tailed *p*-value < 0.05 for Egger regression is considered as the presence of publication bias.

### AMH Assay methods

Two ELISA kits are available worldwide to evaluate AMH levels which are manufactured by Diagnostic Systems Laboratories, Inc. (DSL) and Immunotech [17]; both of which are subsidiaries of Beckman Coulter, Inc. Recently, a new generation of ELISA kit called "AMH Gen II ELISA" has been introduced by Beckman Coulter which somehow replaced the previous kits. Studies have shown that the antibodies used in the AMH Gen II ELISA kit are similar to the DSL with the IoT assay kit standards [13]. The sensitivity and coefficients of inter and intra-assay variation of the kits are shown in Table 1.

**Table 1:** AMH assay kits

Kit Name	Sensitivity	Coefficients of variation intra-assay	Coefficients of variation inter-assay
Active MIS/AMH ELISA (DSL, Webster, TX, USA),	0.04 pmol/l	< 4.6%	< 8.0%
EIA AMH/MIS (IOT, Marseille, France)	0.7 pmol/l	< 12.3%	< 14.2%
AMH Gen II (Beckman Coulter, Chaska, MN, USA)	0.57 pmol/l	< 5.4%	< 5.6%

*DSL* Diagnostic Systems Laboratories, *IOT* Immunotech

### Statistical analysis

Standard mean differences (Std. mean difference) with 95% confidence intervals [18] for continuous outcomes were used to evaluate the pooled effects. Specific outcome data were included in the analysis if they met the required criteria. The data were extracted for analysis wherever attainable and when the trial reports were inadequate or missing, the authors were contacted via email for further information. For the meta-analysis, the number of participants and AMH levels were recorded during a menstrual cycle. The heterogeneity of the included studies was defined by a visual investigation of the outcome tables and the χ2 test. The I^2^ test (represents the rate of variability in effect estimates that is due to heterogeneity rather than sampling error [19]) was used to quantify any apparent discrepancy. An I^2^ value > 50% may represent considerable heterogeneity. Data management and statistical analysis were undertaken using the Comprehensive Meta-Analysis Software (CMA) v.3.3.

## Results

### Study selection and characteristics

**Fig. 1:**
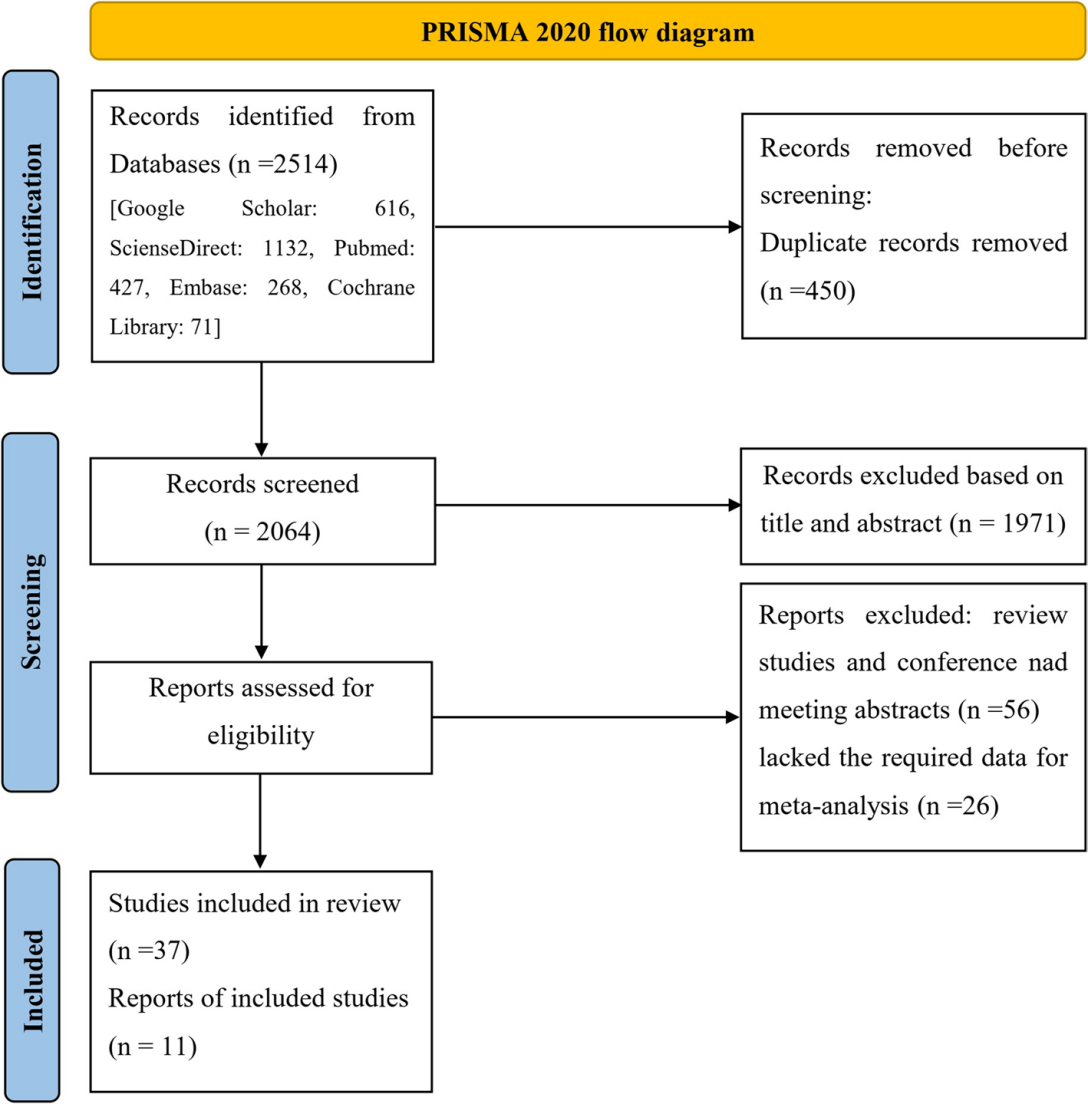
Flow-chart of the study selection process

The preparatory search using the above keywords resulted in 2514 among which 450 studies were excluded due to duplication. After reading the title and abstract of the studies, 1971 papers were also excluded. The full text of 93 remained studies was further evaluated and 56 review studies or conference/meeting abstracts were excluded. Among 37 remained articles, 28 of them lacked the required data for meta-analysis. After several times of correspondence with the authors, we obtained the required data for two studies [20, 21]. Therefore, the data of 26 articles were not used in the analysis, but their findings were discussed in the present study. A total of 11 articles were finally selected for the meta-analysis [17, 20–29]. The search procedure is shown as a flowchart in Fig. 1. The principal characteristics and quality features of the 11 included studies are shown in Table 2.

**Table 2:** Characteristics of included studies

Author(s) (year), Location	Age of participants (year)	Groups (menstrual cycle phases)	Number of participants	Method of measurement	Results
Cook et al. (2000), USA [22]	22–35	F/O/L	20	ELISA	Significant difference among follicular (1.4 ± 0.9 ng/ml), ovulatory (1.7 ± 1.1 ng/ml) and luteal (1.4 ± 0.9 ng/ml) phases [*p* < 0.008]
Elder-Geva et al. (2005), Israel [17]	< 38	F/L	56	IOT	No comparison between the phases
La Marca et al. (2006), Italy [23]	18–24	F/L	12	IOT	No significant difference between follicular (3.9 ± 1.3 ng/ml) and luteal (3.4 ± 1.1 ng/ml) phases [*p* > 0.05]
Elgindy et al. (2008), Egypt [24]	≤ 37	F/O/L	33	IOT	No significant difference among follicular (1.4 ± 1.1 ng/ml), ovulatory (1.43 ± 1.08 ng/ml) and luteal (1.35 ± 1.02 ng/ml) phases [*p* > 0.05]
Streuli et al. (2008), Switzerland [25]	24.1 ± 3.5	F/L	10	IOT	No significant difference between follicular (4.4 ± 1.2 ng/ml) and luteal (4.3 ± 2.29 ng/ml) phases [*p* > 0.05]
Robertson et al. (2011), Australia [26]	21–35	F/L	18	DSL	No significant difference between follicular (4.11 ± 2.49 ng/ml) and luteal (3.66 ± 2.43 ng/ml) phases [*p* > 0.05]
Deb et al. (2013), UK [27]	18–35	F/O/L	35	DSL	Significant difference among follicular (2.6 ± 1.39 ng/ml), ovulatory (2.61 ± 1.42 ng/ml) and luteal (2.92 ± 1.66 ng/ml) phases [*p* = 0.041]
Kissell et al. (2014), USA [20]	18–44	F/O/L	259	Gen II	Significant difference between follicular (2.05 ± 2.09 ng/ml) and ovulatory phases (1.79 ± 1.08 ng/ml) [*p* < 0.01] Significant difference between ovulatory (1.79 ± 1.08 ng/ml) and luteal phases (1.93 ± 1.84 ng/ml) [*p* = 0.01] Significant difference between follicular (2.05 ± 2.09 ng/ml) and luteal phases (1.93 ± 1.84 ng/ml) [*p* = 0.05]
Pankhurst et al. (2016), New Zealand [21]	18–30	F/O/L	11	Gen II	No significant difference among follicular (6.412 ± 3.7 ng/ml), ovulatory (6.2 ± 2.96 ng/ml) and luteal (5.8 ± 2.83 ng/ml) phases [*p* > 0.05]
Melado et al. (2018), Spain [28]	18–38	F/O/L	22	Elecsys® AMH automated assay (Roche®)	Significant difference among follicular (2.93 ± 1.74 ng/ml), ovulatory (2.91 ± 1.82 ng/ml) and luteal (2.95 ± 1.61 ng/ml) phases [*p* < 0.05]
Gorkem et al. (2019), Turkey [29]	18–38	F/L	257	Gen II	Significant difference between follicular (4.3 ± 3.9 ng/ml) and luteal phases (3.5 ± 3.1 ng/ml) [*p* < 0.001]

*F* follicular, *O* ovulatory, *L* luteal

### Quality assessment

The methodological quality of the qualified studies was low to moderate. nine studies were considered as presenting low risk and two as a moderate risk of bias (shown in Table 3). Geographic location, sample size, and measurement methods were all considered to be heterogeneous in the studies. Given the low statistical heterogeneity, a fixed-effects model was used.

**Table 3:** Risk of bias assessment according to Joanna Briggs Institute critical appraisal tool for prevalence studies

	**Criteria**										
1	Was the sample frame appropriate to address the target population?										
2	Were study participants sampled in an appropriate way?										
3	Was the sample size adequate?										
4	Were the study subjects and the setting described in detail?										
5	Was the data analysis conducted with sufficient coverage of the identified sample?										
6	Were valid methods used for the identification of the condition?										
7	Was the condition measured in a standard, reliable way for all participants?										
8	Was there appropriate statistical analysis?										
9	Was the response rate adequate, and if not, was the low response rate managed appropriately?										
	Quality Rating										
	Rater #1 R.K										
	Rater #2 M.P										
	Rater #3 Y.R.R										
**Studies**	**1**	**2**	**3**	**4**	**5**	**6**	**7**	**8**	**9**	**Total (% of “yes”)**	**Risk of bias**
Cook et al. 2000 [22]	Y	U	Y	N	Y	Y	U	Y	Y	66.67	Moderate
Elder-Geva et al. 2005 [17]	Y	Y	Y	Y	Y	Y	Y	N	Y	88.89	Low
La Marca et al. 2006 [23]	Y	Y	N	Y	Y	Y	Y	Y	N	77.78	Low
Elgindy et al. 2008 [24]	Y	Y	Y	Y	Y	Y	Y	Y	Y	100	Low
Streuli et al. 2008 [25]	Y	Y	Y	Y	Y	N	U	Y	Y	77.78	Low
Robertson et al. 2011 [26]	Y	Y	Y	N	Y	Y	Y	N	Y	77.78	Low
Deb et al. 2013 [27]	Y	Y	Y	Y	Y	Y	Y	Y	Y	100	Low
Kissel et al. 2014 [20]	Y	Y	Y	Y	Y	Y	Y	Y	Y	100	Low
Pankhurst et al. 2016 [21]	Y	Y	N	N	Y	Y	U	Y	Y	66.67	Moderate
Melado et al. 2018 [28]	Y	Y	Y	Y	Y	Y	Y	Y	Y	100	Low
Gorkem et al. 2019 [29]	Y	Y	Y	Y	Y	Y	Y	Y	Y	100	Low

*N* no, *U* unclear, *Y* yes, *NA* Not applicable

### Risk of bias

The Egger’s and Begss test provided no evidence of publication bias when analyses were performed for AMH levels fluctuation between follicular and luteal phase (*p* = 0.476 and 0.243, respectively).

### Main analysis

#### Follicular vs. ovulatory phases

**Fig. 2:**
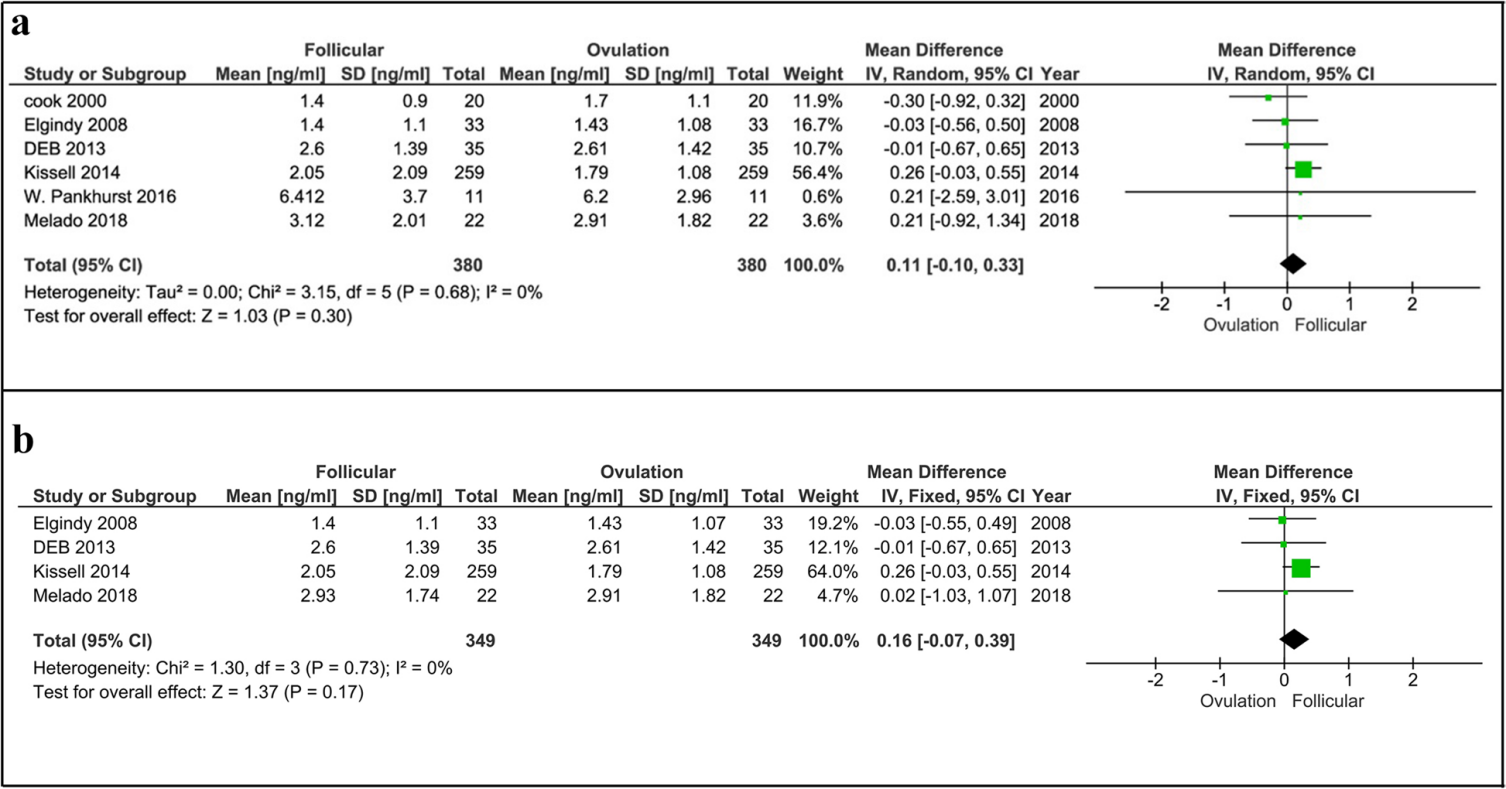
Forest plot of AMH comparison between follicular and ovulatory phases (**a**), including all studies and (**b**), including studies with low risk of bias. AMH, Anti-Müllerian hormone

A total of 11 studies involving 733 women that had evaluated fluctuation of serum AMH levels in follicular, ovulatory, and luteal phases were eligible for the meta-analysis. Six out of 11 articles involving 380 women examined AMH change during the follicular and ovulatory phases and three of them acknowledged that there was no significant difference between the phases [21, 24, 28]. The results of our meta-analysis also showed that the AMH variation in the follicular compared to ovulatory phases was not significant (*p* = 0.30, Fig. 2a) [6 studies, Fixed effects, IV, (95% Cl) = 0.11 [-0.10, 0.33]. We also analyzed the variation after skipping data of two studies with moderate risk [21, 22] and our results again showed no significant difference between the phases (*p* = 0.17, Fig. 2b) [4 studies, Fixed effects, IV, (95% Cl) = 0.16 [-0.07, 0.39]. However, Kissel et al. [20] found that the amount of AMH in the follicular phase was higher than in the ovulatory phase, which was consistent with the Melado et al. [28] findings. Moreover, Cook et al. [22] reported that AMH levels were higher in the ovulatory phase than in the follicular phase.

#### Ovulatory vs. luteal phases

**Fig. 3:**
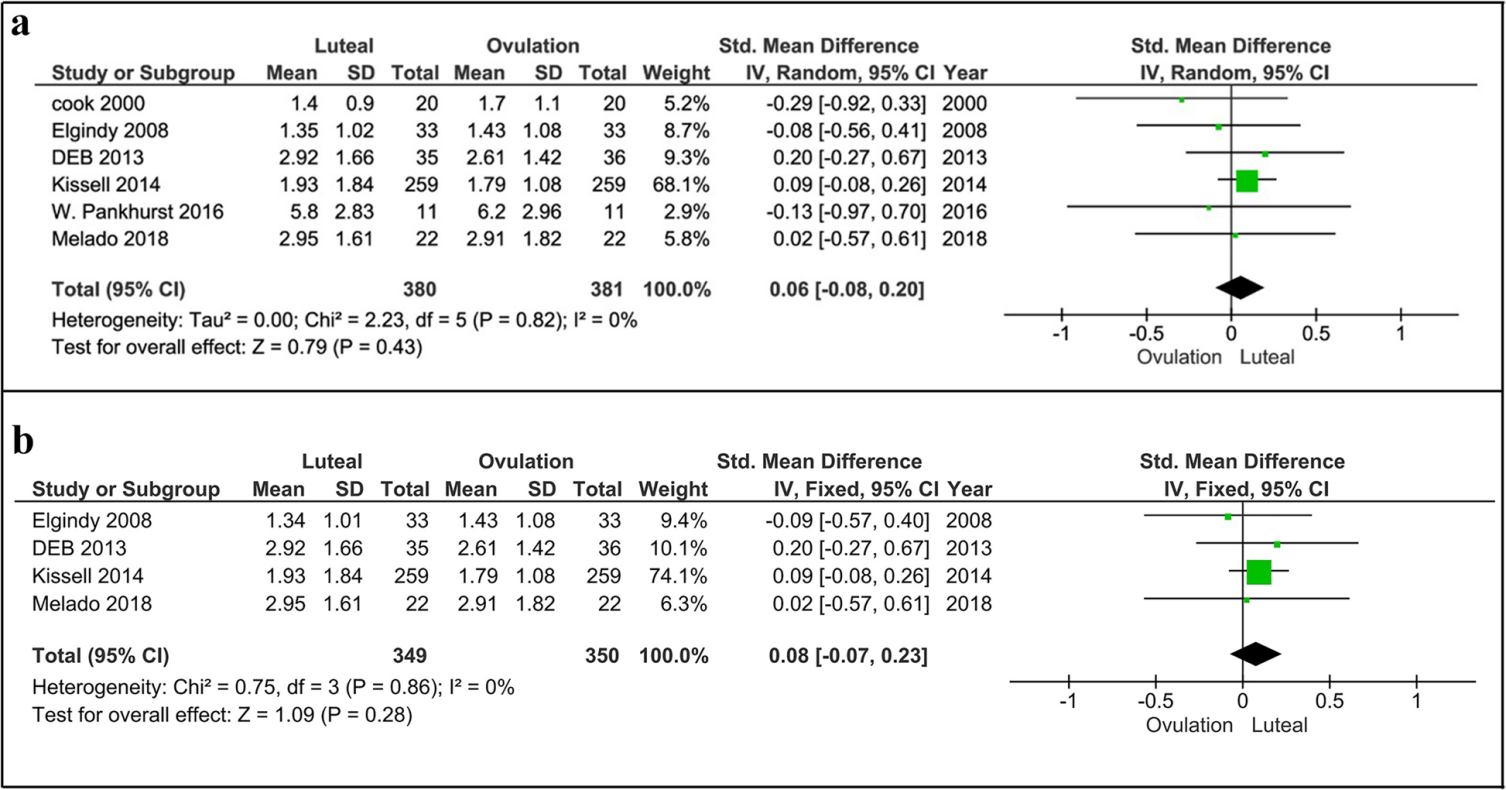
Forest plot of AMH comparison between luteal and ovulatory phases (**a**), including all studies and (**b**), including studies with low risk of bias. AMH, Anti-Müllerian hormone

Comparing the ovulatory and luteal phases, Pankhurst et al. [21] and Elgindy et al. [24] showed no significant changes in the serum levels of AMH, while three studies found higher levels of AMH in the luteal phase than ovulatory phase [20, 27, 28]. Cook et al. [22] also reported higher levels of AMH in the ovulatory phase compared to the luteal phase. The meta-analysis also demonstrated that there was no significant difference in AMH levels between the luteal and ovulatory phases (*p* = 0.43, Fig. 3a) [6 studies, Fixed effects, IV, (95% Cl) = 0.06 [-0.08, 0.20]. Re-analyzing the data after excluding findings of Cook et al. [22] and Pankhurst et al. [21] studies with moderate risk demonstrated no significant difference in AMH levels between luteal and ovulatory phases (*p* = 0.28, Fig. 3b) [4 studies, Fixed effects, IV, (95% Cl) = 0.08 [-0.07, 0.23].

#### Follicular vs. luteal phases

**Fig. 4:**
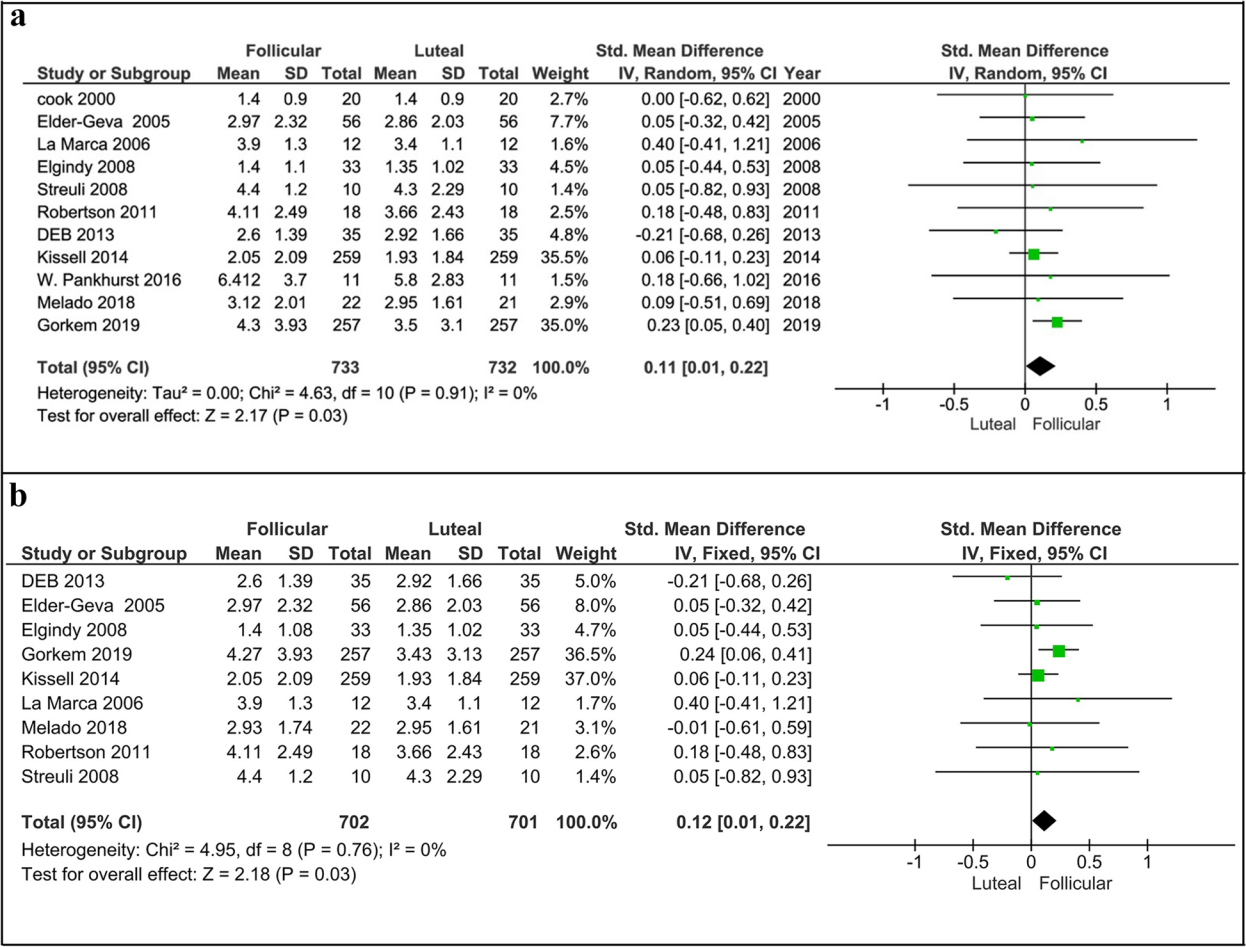
Forest plot of AMH comparison between follicular and luteal phases (**a**), including all studies and (**b**), including studies with low risk of bias. AMH, Anti-Müllerian hormone

Three out of 11 studies acknowledged that serum AMH levels were significantly higher in the follicular phase than in the luteal phase [20, 28, 29]. In this regard, Kissel et al. [20] and Gorkem et al. [29] with the highest sample size among the analyzed studies (259 and 257 women, respectively) have reported higher levels of AMH in the follicular phase compared to the luteal phase. In contrast, Deb et al. [27] have evaluated AMH levels in 35 women and showed that the levels of this hormone were significantly higher in the luteal phase than in the follicular. On the other hand, six studies with a total sample size of 104 women have demonstrated that AMH levels were not significantly different between the follicular and luteal phases [21–26]. Elder-Geva et al. [17] also evaluated AMH levels during the menstrual cycle but they did not statistically compare its levels between the cycle phases; However, we included the data of this study in the meta-analysis. The meta-analysis revealed that serum levels of AMH‌ significantly varied (about 11.5%) throughout the cycle and its levels were statistically higher in the follicular phase than luteal phase (*p* < 0.05, Fig. 4a) [11 studies, Fixed effects, IV, (95% Cl) = 0.11 [0.01, 0.21]. Even excluding the studies with moderate risk [21, 22] did not change the final result and we again found a significant difference in AMH levels between follicular and luteal phases (*p* = 0.03, Fig. 4b) [9 studies, Fixed effects, IV, (95% Cl) = 0.12 [0.01, 0.22].

### AMH Assay methods

**Fig. 5:**
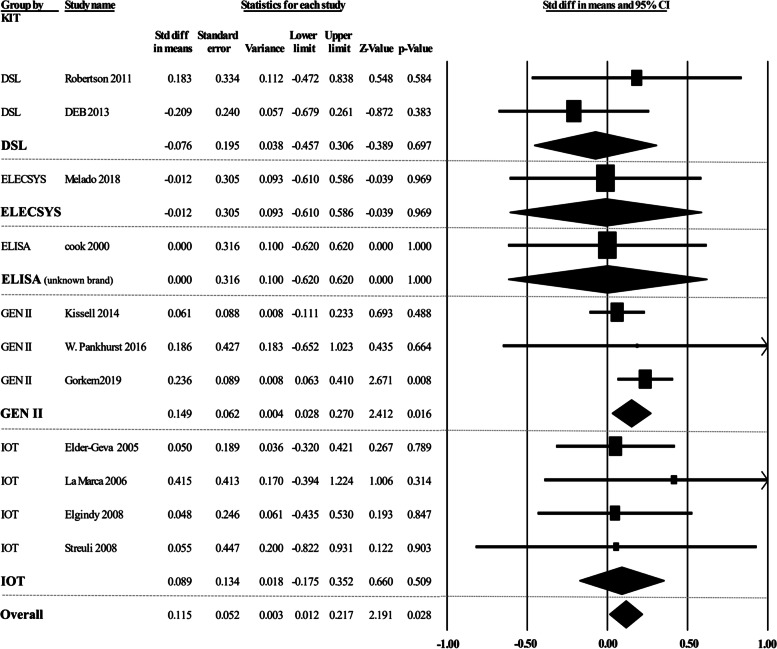
Forest plot of commercial kits comparison applied to evaluate AMH levels. AMH, Anti-Müllerian hormone; ELISA, enzyme-linked immunosorbent assay; DSL, Diagnostic Systems Laboratories; IOT, Immunotech

A sub-analysis was done for different commercial kits (Fig. 5). Our results indicated that there was no significant variation in the AMH levels among different studies that used the same commercial kits, including DSL and IOT (*p* > 0.05). However, we found a significant difference among three studies [20, 21, 29] that applied the GEN II kit for AMH evaluation (*p* = 0.016). This difference might be due to the heterogeneity of the study population as Gorkem et al. [29] recruited infertile women while Kissell et al. [20] and Pankhurst et al. [21] collected the samples from women with a regular menstrual cycle. Overall, AMH variation was significantly different among various commercial kits (*p* = 0.028) showing that the method of assay can be also important in the result.

## Discussion

### Finding of the review

One of the advantages of AMH measuring compared to other hormones such as LH or FSH is that its levels have the slightest fluctuation during the menstrual cycle and therefore can be evaluated on any day of the menstrual cycle. However, by improving the sensitivity and accuracy of the measurement methods, studies on the stability of AMH levels during the menstrual cycle have yielded conflicting results that make it challenging to clinically interpret the serum levels of AMH. The present systematic review and meta-analysis showed significant fluctuations in the serum levels of AMH during the menstrual cycle. Meta-analysis of pooled data showed that amount of AMH in the follicular phase is higher than in the luteal phase, whether this significant fluctuation might influence clinical practice concerning the timing of AMH measurement. It has been reported that gonadotropins may be involved in stimulating gonadotropin-dependent follicles and consequently AMH levels change [30]. Moreover, it has been shown that AMH changes during a menstrual cycle might be due to biological alteration and atypical AMH isoforms [31]. The underlying mechanism(s) that cause fluctuation in AMH levels during a menstrual cycle has not been fully addressed. Nevertheless, this hormone is mostly expressed in growing preantral and antral follicles [32]. On the other hand, it has been reported that the total number of antral follicles decreases during the cycle from follicular to menstrual phase, possibly due to the negative effect of luteinization on granulosa cells [27]. Therefore, higher levels of AMH in the follicular phase compared to the luteal phase could be due to a reduction in the follicles producing this hormone. Another potential reason for such difference could be due to biological variations of AMH and existing of unusual isoforms [31]. Moreover, it has been documented that gonadotropins variation during the menstrual cycle affects gonadotropin-dependent follicles and consequently the AMH level [30, 33].

Since serum AMH is clinically important for the evaluation of ovarian reserve [34] and also its elevated level (> 3–4 ng/ml) is associated with PCOS [35], the AMH fluctuation during the menstrual cycle might affect the interpretation of the laboratory result. More importantly, in infertile patients, serum AMH is used to obtain prognostic information regarding the chance of successful ovarian stimulation as well as pregnancy [36]. Although it should be noted that the standardized mean difference between follicular and luteal phases was 0.12 which is equated to effect sizes of small. On the other hand, the normal range of serum AMH levels is low, and the slightest change in it, which can be due to the phase of measurement, can place women in different groups in terms of ovarian reserve, including normal, low, and very low. This variation can be more important in women whose AMH is near the cut-off value. Gynecologists make important decisions often based on serum AMH levels such as the dose of gonadotropins for ovarian stimulation in ART cycles [37]. Accurate AMH levels can better reflect the growing follicle status in the ovary and the expectation for oocyte retrieval following hormone stimulation. Therefore, it might be suggested that evaluating AMH level at right time in combination with antral follicle count could better help gynecologists to decide on the appropriate dosage of gonadotropins for ovarian stimulation.

### Strength of this review

Based on our knowledge, this is the first systematic review and meta-analysis study which included longitudinal papers and also systematically analyze all the available data that examined AMH fluctuations during a menstrual cycle. One of the strengths of the current study is the inclusion of a large number of participants that can bring the results closer to reality. In addition, this meta-analysis includes only studies that have examined AMH levels throughout one menstrual cycle in order to clarify the intercycle variation. Besides, we analyzed the data phase by phase and significant changes were examined for each phase of the menstrual cycle. Moreover, the target population was all of the reproductive age at which the AMH level is relatively more stable.

### Limitation

The division of each phase was not performed in some included studies that challenged the uniformity of the selected days for each phase. Also, clinically significant heterogeneity including inattention in selecting and defining the groups of poor, normal, and high responders, differences in the method of testing and assay kit for serum AMH levels, as well as differences in statistical tests in the included studies were some of the limitations ahead.

## Conclusion

This study indicates that the serum level of AMH which is considered one of the stable and reliable factors for ovarian reserve evaluation has significant fluctuation during a menstrual cycle. Therefore, it may be specified on which day of the menstrual cycle (or at least which phase of the cycle) the patient can be referred for AMH measurement, and also the physician may consider the time of sampling in the interpretation of the lab results. Since the range of serum AMH levels is low, especially in women over 35 years of age, the standardized mean difference of 0.12 between follicular and luteal phases could put a woman in a different group of ovarian reserve and therefore affect the physician’s therapeutic strategy. For example, women aged < 35 years old with serum AMH levels of 1.1 ng/ml are considered normal while 0.9 ng/ml is assumed as a low ovarian reserve. Moreover, in patients with low and very low AMH levels, the decision of gynecologists can be changed regarding the dose of ovarian stimulation or even applying platelet-rich plasma therapy. The results of this study also showed that there is no significant difference in AMH levels between the ovulatory phase in comparison with follicular and luteal phases; therefore, it can be postulated that measurement of AMH levels in the ovulation phase might better reflect the ovarian reserve. Moreover, the combination of antral follicle count and AMH levels could able clinicians to better decide on the dosage of gonadotropins for ART cycles.

## Data Availability

The datasets used and/or analyzed during the current study are available from the corresponding author on reasonable request.

## Abbreviations

AFC: Antral follicle counts
AMH: Anti-Müllerian hormone
ART: Assisted reproductive technology
COH: Controlled ovarian hyperstimulation
ELISA: Enzyme-linked immunosorbent assay
PCOS: Polycystic ovaries syndrome
POF: Premature ovarian failure
PRISMA: Preferable Reporting Items for Systematic Reviews and Meta-Analyses

## References

[1] Lee MM, Donahoe PK, Hasegawa T, Silverman B, Crist GB, Best S (1996). Mullerian inhibiting substance in humans: normal levels from infancy to adulthood. J Clin Endocrinol Metab.

[2] Jayaprakasan K, Campbell B, Hopkisson J, Johnson I, Raine-Fenning N (2010). A prospective, comparative analysis of anti-Müllerian hormone, inhibin-B, and three-dimensional ultrasound determinants of ovarian reserve in the prediction of poor response to controlled ovarian stimulation. Fertil Steril.

[3] La Marca A, Sighinolfi G, Radi D, Argento C, Baraldi E, Artenisio AC (2010). Anti-Müllerian hormone (AMH) as a predictive marker in assisted reproductive technology (ART). Hum Reprod Update.

[4] Van Rooij I, Broekmans F, Te Velde E, Fauser B, Bancsi L, De Jong F (2002). Serum anti-Müllerian hormone levels: a novel measure of ovarian reserve. Hum Reprod.

[5] Yassin MM, Sharif FA, Laqqan MM (2013). Anti-mullerian hormone as a predictor of ovarian reserve and ovarian response in IVF women from Gaza strip. Iranian Journal of Reproductive Medicine.

[6] Heidar Z, Bakhtiyari M, Mirzamoradi M, Zadehmodarres S, Sarfjoo F, Mansournia M (2015). Prediction of different ovarian responses using anti-Müllerian hormone following a long agonist treatment protocol for IVF. J Endocrinol Invest.

[7] La Marca A, Volpe A (2006). Anti-Müllerian hormone (AMH) in female reproduction: is measurement of circulating AMH a useful tool?. Clin Endocrinol.

[8] Bersinger NA, Wunder D, Birkhäuser MH, Guibourdenche J (2007). Measurement of anti-mullerian hormone by Beckman Coulter ELISA and DSL ELISA in assisted reproduction: differences between serum and follicular fluid. Clin Chim Acta.

[9] Fréour T, Mirallié S, Bach-Ngohou K, Denis M, Barrière P, Masson D (2007). Measurement of serum anti-Müllerian hormone by Beckman Coulter ELISA and DSL ELISA: comparison and relevance in assisted reproduction technology (ART). Clin Chim Acta.

[10] Hehenkamp WJK, Looman CWN, Themmen APN, De Jong FH, Te Velde ER, Broekmans FJM (2006). Anti-Müllerian hormone levels in the spontaneous menstrual cycle do not show substantial fluctuation. J Clin Endocrinol Metab.

[11] Streuli I, Fraisse T, Chapron C, Bijaoui G, Bischof P, De Ziegler D (2009). Clinical uses of anti-Müllerian hormone assays: pitfalls and promises. Fertil Steril.

[12] Nelson S, La Marca A (2011). The journey from the old to the new AMH assay: how to avoid getting lost in the values. Reprod Biomed Online.

[13] Kumar A, Kalra B, Patel A, Mcdavid L, Roudebush WE (2010). Development of a second generation anti-Müllerian hormone (AMH) ELISA. J Immunol Methods.

[14] Moher D, Liberati A, Tetzlaff J, Altman DG (2010). Preferred reporting items for systematic reviews and meta-analyses: the PRISMA statement. Int J Surg.

[15] JBI critical appraisal checklist for studies reporting prevalence data. https://joannabriggs.org/sites/default/files/2019-05/JBI_Critical_Appraisal-Checklist_for_Prevalence_Studies2017_0.pdf.

[16] Munn Z, Moola S, Lisy K, Riitano D, Tufanaru C (2015). Methodological guidance for systematic reviews of observational epidemiological studies reporting prevalence and cumulative incidence data. Int J Evid Based Healthc.

[17] Eldar-Geva T, Ben-Chetrit A, Spitz IM, Rabinowitz R, Markowitz E, Mimoni T (2005). Dynamic assays of inhibin B, anti-Mullerian hormone and estradiol following FSH stimulation and ovarian ultrasonography as predictors of IVF outcome. Hum Reprod.

[18] Yáñez-Mó M, Siljander PRM, Andreu Z, Zavec AB, Borràs FE, Buzas EI (2015). Biological properties of extracellular vesicles and their physiological functions. Journal of extracellular vesicles.

[19] Higgins JP, Thompson SG, Deeks JJ, Altman DG (2003). Measuring inconsistency in meta-analyses Bmj.

[20] Kissell KA, Danaher MR, Schisterman EF, Wactawski-Wende J, Ahrens KA, Schliep K (2014). Biological variability in serum anti-Müllerian hormone throughout the menstrual cycle in ovulatory and sporadic anovulatory cycles in eumenorrheic women. Hum Reprod.

[21] Pankhurst MW, Chong YH (2016). Variation in circulating antimüllerian hormone precursor during the periovulatory and acute postovulatory phases of the human ovarian cycle. Fertil Steril.

[22] Cook CL, Siow Y, Taylor S, Fallat ME (2000). Serum mullerian-inhibiting substance levels during normal menstrual cycles. Fertil Steril.

[23] La Marca A, Stabile G, Carducci Artenisio A, Volpe A (2006). Serum anti-Mullerian hormone throughout the human menstrual cycle. Hum Reprod.

[24] Elgindy EA, El-Haieg DO, El-Sebaey A (2008). Anti-Müllerian hormone: correlation of early follicular, ovulatory and midluteal levels with ovarian response and cycle outcome in intracytoplasmic sperm injection patients. Fertil Steril.

[25] Streuli I, Fraisse T, Pillet C, Ibecheole V, Bischof P, De Ziegler D (2008). Serum antimüllerian hormone levels remain stable throughout the menstrual cycle and after oral or vaginal administration of synthetic sex steroids. Fertil Steril.

[26] Robertson DM, Hale GE, Fraser IS, Hughes CL, Burger HG (2011). Changes in serum antimüllerian hormone levels across the ovulatory menstrual cycle in late reproductive age. Menopause.

[27] Deb S, Campbell BK, Clewes JS, Pincott-Allen C, Raine-Fenning NJ (2013). Intracycle variation in number of antral follicles stratified by size and in endocrine markers of ovarian reserve in women with normal ovulatory menstrual cycles. Ultrasound Obstet Gynecol.

[28] Melado L, Lawrenz B, Sibal J, Abu E, Coughlan C, Navarro AT (2018). Anti-müllerian Hormone During Natural Cycle Presents Significant Intra and Intercycle Variations When Measured With Fully Automated Assay. Front Endocrinol.

[29] Gorkem U, Togrul C (2019). Is There a Need to Alter the Timing of Anti-Müllerian Hormone Measurement during the Menstrual Cycle?. Geburtshilfe Frauenheilkd.

[30] Tran ND, Cedars MI, Rosen MP (2011). The role of anti-müllerian hormone (AMH) in assessing ovarian reserve. J Clin Endocrinol Metab.

[31] Robertson DM, Kumar A, Kalra B, Shah S, Pruysers E, Brink HV (2014). Detection of serum antimüllerian hormone in women approaching menopause using sensitive antimüllerian hormone enzyme-linked immunosorbent assays. Menopause.

[32] Ueno S, Kuroda T, Maclaughlin DT, Ragin RC, Manganaro TF, Donahoe PK (1989). Mullerian inhibiting substance in the adult rat ovary during various stages of the estrous cycle. Endocrinology.

[33] Hagen CP, Sørensen K, Anderson RA, Juul A (2012). Serum levels of antimüllerian hormone in early maturing girls before, during, and after suppression with GnRH agonist. Fertil Steril.

[34] Cui L, Qin Y, Gao X, Lu J, Geng L, Ding L (2016). Antimüllerian hormone: correlation with age and androgenic and metabolic factors in women from birth to postmenopause. Fertil Steril.

[35] Dumont A, Robin G, Catteau-Jonard S, Dewailly D (2015). Role of Anti-Müllerian Hormone in pathophysiology, diagnosis and treatment of Polycystic Ovary Syndrome: a review. Reprod Biol Endocrinol.

[36] Shrikhande L, Shrikhande B, Shrikhande A (2020). AMH and its clinical implications. The Journal of Obstetrics and Gynecology of India.

[37] Bungum L, Tagevi J, Jokubkiene L, Bungum M, Giwercman A, Macklon N (2018). The impact of the biological variability or assay performance on AMH measurements: A prospective cohort study with AMH tested on three analytical assay-platforms. Front Endocrinol.

